# Editorial: The utilization of bench-to-bedside approaches in pharmacogenomics

**DOI:** 10.3389/fphar.2023.1234219

**Published:** 2023-06-13

**Authors:** Eric Rytkin, Kseniia Kriukova, Natalia Denisenko, Dmitriy Ivashchenko, Michael Zastrozhin, Karin Mirzaev, Dmitry Sychev

**Affiliations:** ^1^ Department of Biomedical Engineering, Northwestern University, Chicago, IL, United States; ^2^ Keck School of Medicine of USC, University of Southern California, Los Angeles, CA, United States; ^3^ Sechenov University, Moscow, Russia; ^4^ Russian Medical Academy of Continuous Professional Education, Moscow, Russia; ^5^ Department of Bioengineering and Therapeutical Sciences of UCSF, University of California San Francisco, San Francisco, CA, United States

**Keywords:** pharmacogenomics, core facilities, bench to bedside translation, clinical application, biomarkers

## Introduction

Pharmacogenetic testing is gaining prominence in clinical practice as evidence-based recommendations, and prospective studies demonstrate the advantages of a personalized genotype-based approach over standard therapy regimens (Weinshilboum and Wang, 2017; Luzum et al., 2021). In recent years, pharmacogenomics has emerged as a promising field that combines pharmacology and genomics to determine how an individual’s genetic makeup influences their response to medications. This personalized medicine approach can potentially revolutionize patient care by optimizing drug selection and dosage based on individual genetic profiles. A bench-to-bedside approach is crucial to translate pharmacogenomic research findings into clinical practice. This editorial explores the utilization of bench-to-bedside approaches in pharmacogenomics and features different aspects of them—from organizing pharmacogenetic laboratories within the academic infrastructure of educational and research institutions to the selection of Predictive Biomarkers.

## The demand for new technologies

Today, pharmacogenetic testing is becoming more and more affordable. The price of genotyping is decreasing every year. There is a growing demand from patients for effective and safe pharmacotherapy. But doctors’ awareness of the possibilities of pharmacogenetic testing remains low: according to various estimates, only up to 30% of specialists are familiar with this technology (Rahawi et al., 2020; Muflih et al., 2021). Consequently, the work on the implementation of pharmacogenetic testing is in demand. It is important not only to educate physicians about the possibilities of personalized pharmacotherapy but also to campaign among patients. Although there are genetic tests on the market that have no proven benefit, it is important to introduce people to new technologies. It is necessary to overcome the psychological barrier that slows down the use of genetic tests. Typically, patients are wary of genome analysis (McCarthy et al., 2020). It is important to include pharmacogenetic testing in clinical guidelines. This will lead to more active prescribing of single nucleotide polymorphism analysis. To date, CPIC and DPWG have made numerous suggestions for personalized pharmacotherapy selection. But the practical application will be limited until pharmacogenetic testing is included in clinical guidelines for the treatment of diseases.

The Organization of Pharmacogenetic Laboratories in Academic Institutions:

While commercial laboratories offer accessibility and scalability, they often lack expertise in marker selection, test prescription strategies, and result interpretation. This results in a lack of credibility among physicians, clinic leaders, health system representatives, and patients (Luzum et al., 2021). To overcome these limitations, organizing pharmacogenetic laboratories within the academic infrastructure of educational and research institutions is a promising approach. This organizational form provides expert support, including marker selection and result interpretation, and offers extensive training programs for healthcare professionals and patients. Academic laboratories can also establish well-described biobanks, collect pharmacogenetic and clinical data, and secure multiple funding sources through research and grant projects.

In academic institutions, Pharmacogenomics laboratories of such a kind are usually organized in the form of Core Facilities. These core facilities usually include equipment for nucleic acid extraction and quantitative and qualitative analysis and may employ various methods such as polymerase chain reaction or high-throughput sequencing (Henslee and Telgenhoff, 2019). Additionally, access to pharmacokinetic laboratory and bioinformatics services can be incorporated into the laboratory structure. Providing access to the pharmacogenetic core facility for other university departments allows collaborative research studies and grant programs (Table 1). Effective laboratory operation relies on financial statements, close interaction with the finance department, and a well-organized procurement system.

**TABLE 1:** The summary of the collaborative studies conducted in the Russian Medical Academy of Continuous Professional Education. This features the collaborations between Clinical Departments and the Pharmacogenomics Center.

Department	Condition/Drug	Article
Department of Clinical Pharmacology and Therapy	Hypertension/Losartan	Sinitsina II, Boyarko AV, Temirbulatov II, et al.*CYP2C9* gene polymorphisms influence on antihypertensive effectiveness and hypouricemic effect of losartan among patients with arterial hypertension: an observational study [published online ahead of print, 2022 December 29]. *Drug Metab Pers Ther*. 2022;10.1515/dmpt-2022-0115. doi:10.1515/dmpt-2022-0115
Department of Urgent and General Surgery	Post-op Pain Management/Ketorolac, Tramadol	Muradian AA, Sychev DA, Blagovestnov DA, et al. The effect of*CYP2D6* and *CYP2C9* gene polymorphisms on the efficacy and safety of the combination of tramadol and ketorolac used for postoperative pain management in patients after video laparoscopic cholecystectomy. *Drug Metab Pers Ther*. 2021;371):27-34. Published 2021 July 12. doi:10.1515/dmpt-2021-0112
Ophthalmology Department	Glaucoma/Timolol	Moshetova LK, Soshina MM, Turkina KI, et al. Effect ofCYP2D6*4, CYP2D6*10 polymorphisms on the safety of treatment with timolol maleate in patients with glaucoma [published online ahead of print, 2022 August 24]. *Drug Metab Pers Ther*. 2022;10.1515/dmpt-2022-0117. doi:10.1515/dmpt-2022-0117
Interventional Cardiology Department	Atrial Fibrillation/Rivaroxaban	Rytkin E, Bure IV, Bochkov PO, et al. MicroRNAs as novel biomarkers for rivaroxaban therapeutic drug monitoring.*Drug Metab Pers Ther*. 2021;371):41-46. Published 2021 August 13. doi:10.1515/dmpt-2021-0118
Department of Radiotherapy and Radiology	Thyroid Cancer/Radioiodine	Denisenko NP, Shuev GN, Mukhamadiev RH, et al. Genetic markers associated with resistance to radioiodine therapy in thyroid cancer patients: Prospective cohort study. J Mod Oncol. 2022;243):345-350. doi: 10.26442/18151434.2022.3.201867
Department of Clinical Pharmacology and Therapy	Hypertension/Enalapril	Sychev IV, Denisenko NP, Kachanova AA, et al. Pharmacogenetic predictors of development of secondary to enalapril dry cough in hypertensive patients [published online ahead of print, 2023 May 19]. *Drug Metab Pers Ther*. 2023;10.1515/dmpt-2023-0008. doi:10.1515/dmpt-2023-0008
Department of Infectious Diseases	COVID-19/Remdesivir	Temirbulatov II, Kryukov AV, Mirzaev KB, et al. The Effect of Carriage of CYP3A53 and CYP3A422 Polymorphic Variants on the Safety of Remdesivir Therapy in Patients with COVID-19. Antibiot Khimioter. 2022;67 (45145):45-50. PMID: covidwho-2245708
Interventional Cardiology Department	Acute Coronary Syndrome/Clopidogrel, Ticagrelor	Rytkin E, Mirzaev K, Bure I, et al. MicroRNAs as Novel Biomarkers for P2Y12 - Inhibitors Resistance Prediction.*Pharmgenomics Pers Med*. 2021;14:1575-1582. Published 2021 December 2. doi:10.2147/PGPM.S324612
Interventional Cardiology Department	Atrial Fibrillation/Calcium Channel Blockers	Sychev D, Mirzaev K, Cherniaeva M, et al. Drug-drug interaction of rivaroxaban and calcium channel blockers in patients aged 80 years and older with nonvalvular atrial fibrillation.*Drug Metab Pers Ther*. 2020;353):10.1515/dmpt-2020-0127. Published 2020 September 4. doi:10.1515/dmpt-2020-0127
Psychiatry Department	Psychotic Disorder/Haloperidol	Parkhomenko AA, Zastrozhin MS, Pozdnyakov SA, et al. Association of CYP2D6*4 Polymorphism with the Steady-State Concentration of Haloperidol in Patients with Alcohol-Induced Psychotic Disorders.*Psychopharmacol Bull*. 2022;524):52-60

## Integrating preventive medicine and personalized medicine

Preventive medicine and personalized medicine aim to provide better-individualized healthcare based on unique patient factors and biomarkers, including genetic factors. The research on predictive biomarkers may vary from pharmacogenomics of antidepressant drugs (Kee et al.) to acute coronary syndrome management (Azzahhafi et al.) and antithrombotic therapy (Asiimwe et al.). (Asiimwe et al.; Azzahhafi et al.; Kee et al.) By identifying genetic variations as predictive biomarkers, pharmacogenomic information on the optimal medication can be obtained, potentially improving patient outcomes. Similar approaches can be applied to other areas, such as neurodegenerative and neuroinflammatory disorders.

### Utilizing bench-to-bedside approaches in pharmacogenomics

To effectively utilize bench-to-bedside approaches in pharmacogenomics, it is crucial to acquire knowledge of predictive biomarkers and study them as both predictive biomarkers and pharmacogenomic tools. Cross et al. discussed the concept of polygenic risk scores (PRS) as an overview of predictive biomarkers in personalized medicine (Cross et al.). PRS is a statistical approach that combines information from multiple genetic variants to estimate an individual’s genetic risk for a particular condition or response to treatment. The integration of PRS into pharmacogenomics has the potential to enhance the predictive power and precision of personalized medicine approaches.

Cross et al. highlighted that PRS can be derived from genome-wide association studies (GWAS) and applied to various medical conditions, including cardiovascular diseases, psychiatric disorders, and cancer (Cross et al.). By considering a broader set of genetic markers, PRS can capture the cumulative effect of multiple genetic variants, allowing for more accurate risk assessment and treatment predictions. Additionally, incorporating PRS into pharmacogenomics can help identify individuals who are at a higher risk of adverse drug reactions or poor treatment response, enabling clinicians to personalize medication choices accordingly.

## Conclusion

The utilization of bench-to-bedside approaches in pharmacogenomics holds great promise for advancing personalized medicine. Organizing pharmacogenetic laboratories within academic institutions provides several advantages, including expert support, extensive training programs, and the establishment of well-described biobanks. Integrating predictive biomarkers, such as PRS, into pharmacogenomics enhances the ability to predict treatment response and optimize therapeutic interventions. By embracing these approaches, we can pave the way for a deeper understanding of diseases and the development of targeted therapies that improve patient outcomes.
